# Balance telerehabilitation and wearable technology for people with Parkinson’s disease (TelePD trial)

**DOI:** 10.1186/s12883-023-03403-3

**Published:** 2023-10-13

**Authors:** Carla Silva-Batista, Jennifer L. Wilhelm, Kathleen T. Scanlan, Margaret Stojak, Patricia Carlson-Kuhta, Siting Chen, William Liu, Tomas Nicolás García de la Huerta, Fay B. Horak, Martina Mancini, Laurie A. King

**Affiliations:** 1 Balance Disorders Laboratory, Department of Neurology, Oregon Health & Science University, 3181 SW Sam Jackson Park Rd, Portland, OR OP-3297239 USA; 2 School of Public Health, Oregon Health & Science University, Portland, OR USA; 3 APDM Precision Motion of Clario, Portland, OR USA

**Keywords:** Objective measures, Mobility, Telehealth, Wearable inertial sensors, Parkinson’s disease, Home exercises

## Abstract

**Background:**

Balance impairments, that lead to falls, are one of the main symptoms of Parkinson’s disease (PD). Telerehabilitation is becoming more common for people with PD; however, balance is particularly challenging to assess and treat virtually. The feasibility and efficacy of virtual assessment and virtual treatment of balance in people with PD are unknown. The present study protocol has three aims: I) to determine if a virtual balance and gait assessment (instrumented L-shape mobility test) with wearable sensors can predict a gold-standard, in-person clinical assessment of balance, the Mini Balance Evaluation Systems Test (Mini-BESTest); II) to explore the effects of 12 sessions of balance telerehabilitation and unsupervised home exercises on balance, gait, executive function, and clinical scales; and III) to explore if improvements after balance telerehabilitation transfer to daily-life mobility, as measured by instrumented socks with inertial sensors worn for 7 days.

**Methods:**

The TelePD Trial is a prospective, single-center, parallel-group, single-blind, pilot, randomized, controlled trial. This trial will enroll 80 eligible people with PD. Participants will be randomized at a 1:1 ratio into receiving home-based balance exercises in either: 1) balance telerehabilitation (experimental group, *n* = 40) or 2) unsupervised exercises (control group, *n* = 40). Both groups will perform 12 sessions of exercise at home that are 60 min long. The primary outcome will be Mini-BESTest. The secondary outcomes will be upper and lower body gait metrics from a prescribed task (instrumented L-shape mobility test); daily-life mobility measures over 7 days with wearable sensors in socks, instrumented executive function tests, and clinical scales. Baseline testing and 7 days of daily-life mobility measurement will occur before and after the intervention period.

**Conclusion:**

The TelePD Trial will be the first to explore the usefulness of using wearable sensor-based measures of balance and gait remotely to assess balance, the feasibility and efficacy of balance telerehabilitation in people with PD, and the translation of balance improvements after telerehabilitation to daily-life mobility. These results will help to develop a more effective home-based balance telerehabilitation and virtual assessment that can be used remotely in people with balance impairments.

**Trial registration:**

This trial was prospectively registered on ClinicalTrials.gov (NCT05680597).

**Supplementary Information:**

The online version contains supplementary material available at 10.1186/s12883-023-03403-3.

## Introduction

During the Coronavirus disease (COVID-19) pandemic, millions of people were forced to self-isolate and halt regular exercise and clinical visits. Several studies recently highlighted the impact of the pandemic on people with Parkinson’s disease (PD), including worsening motor symptoms, mood, anxiety, and insomnia [1–3]. Self-isolation for people with PD resulted in clinical balance (Mini Balance Evaluation Systems Test [Mini-BESTest]) scores that worsened in just two months, with increased falls [3]. From (less common) pandemics to (more common) inaccessibility to specialized care, balance telerehabilitation may become an essential aspect of rehabilitation care for people with PD and it is important to fully explore the feasibility and effectiveness.

Successful balance telerehabilitation requires the ability to perform both virtual assessment and virtual treatment. Balance is particularly challenging to assess virtually because a thorough and detailed in-person balance assessment typically involves measuring performance across multiple domains of balance (e.g., anticipatory postural adjustment [APA], postural sway, trunk range of motion, and turning) and with enough challenge to evoke imbalance, but this is difficult to carry out safely with virtual visits. Previous studies have demonstrated the feasibility of virtually assessing fall-related measures such as 5 times sit-to-stand [4], 360-degree rapid-turn-test [4], and motor symptoms by the Movement Disorder Society-sponsored revision of the Unified Parkinson's Disease Rating Scale (MDS-UPDRS) part III [5, 6]. However, a modified version of the MDS-UPDRS part III that does not include rigidity and postural instability as these items can’t be assessed virtually, has been demonstrated to be generally feasible for telehealth visits in PD [5, 7, 8]. Although recent feasibility studies have assessed clinical balance (Berg Balance Scale) [9] and motor symptoms (MDS-UPDRS-III—modified) [6, 9] virtually, before and after telerehabilitation, to the best of our knowledge, no study has assessed multiple domains of balance virtually. To capture multiple domains of balance and gait in one test, we used the Instrumented Stand and Walk Test (ISAW), a short balance and gait task, using wearable inertial sensors [10, 11]. The ISAW test results in metrics from different domains of mobility: postural sway in quiet stance, APA associated with step initiation, quality of turning 180°, and gait characteristics, that have been shown to be independent such as spatial, temporal, and upper body [12, 13]. Thus, it may be feasible to use similar task with wearable inertial sensors during a virtual visit to assess balance and gait using an instrumented L-shape mobility test. In this study, we hypothesize that a virtual visit exam using wearable sensors to quantify balance and gait during a prescribed standing, walking, and turning task (instrumented L-shape mobility test) will predict a gold-standard, in-person assessment of balance (Mini-BESTest).

Although telerehabilitation is becoming more common for people with PD, it remains to be seen whether it is feasible and efficacious for improving balance control [1, 14–17]. One recent systematic review and meta-analysis of randomized controlled trials investigated the effects of home-based virtual reality training and telerehabilitation compared to conventional therapy on balance in people with PD, multiple sclerosis, and stroke [18]. This systematic review and meta-analysis found similar effects of both interventions on balance, which was only assessed by the Berg Balance Scale. Another recent systematic review [19], highlighted the need for high-quality studies of telerehabilitation in PD. Only four studies aimed to explore the effects of telerehabilitation with clinical balance tests, such as the Berg Balance Scale [20, 21], the Activities-specific Balance Confidence scale [21, 22], and Balance Evaluation Systems Test [23]. Of these, only one was a randomized controlled trial that used supervised telerehabilitation in real-time with home-based virtual reality in people with mild-to-moderate PD [21], while the other studies were a case report [22], telerehabilitation without real-time supervision and not home-based [23], and virtual reality delivered in the clinic and at home [20]. Currently, most of the studies have used virtual reality in the telerehabilitation program [18, 19]; however, telerehabilitation in real-time, via videoconferencing, is considered a basic technology [24, 25] and potentially more accessible than virtual reality. In addition, the Clinical Practice Guideline’s recommendation for telerehabilitation is only moderate, while the recommendation for other more established approaches such as balance training, gait training, and task-specific training is high [26]. Thus far, clinical trials of home-based balance telerehabilitation via videoconferencing, supervised in real-time for people with PD are scarce.

Balance telerehabilitation for neurologic patients lacks strong evidence-based trials and it presents unique safety concerns [14] since we need to determine how patients can practice balance exercises in the home during a virtual therapy intervention with minimal risk for falls. There has been great progress in the past decade with regard to implementing motor learning and task specificity for optimal results for balance training [27]. The recent Clinical Practice Guideline recommends implementing aspects of task specificity that target specific impairments in PD such as APA, sensory integration, and challenging gait to improve balance in people with PD [26]. At this point, it is unknown if these Clinical Practice Guideline recommendations can be safely and effectively implemented in a virtual visit setting.

How an exercise program is delivered is important. We previously compared the Agility Boot Camp (ABC) exercise intervention executed via one-on-one, delivery with the physical therapist in the clinic versus at-home, unsupervised exercises (standard of care) [28]. The ABC framework targeted the underlying constraints on balance in people with PD including rigidity, bradykinesia, freezing, inflexible program selection (segmental coordination), impaired sensory integration, and reduced executive function and attention. Not surprisingly, the one-on-one, in-clinic delivery of the ABC program improved balance better than unsupervised at-home ABC [28]. We found that the Mini-BESTest had a large improvement (effect size = 0.86) after 12 sessions of in-person ABC rehabilitation and only a moderate improvement (effect size = 0.67) in the unsupervised, home exercise group [28]. In fact, the unsupervised home group improved very little across all outcome measures, while the in-person group improved the most on function and balance measures [28]. In the current study, we will adapt our original ABC program, designed to target underlying constraints on balance, to be performed virtually with appropriate safety modifications. We hypothesize that a virtual delivery of the ABC exercise program, called Tele-ABC, will be more effective than the unsupervised-ABC program in improving balance. In addition, we will explore the role of cognition, both as a potentially important covariate for successful participation in telerehabilitation and as an outcome measure after rehabilitation since executive and visuospatial functions are linked to balance [29–32], and are very important to daily-life mobility in PD. The Tele-ABC exercise program also uses embedded balance and cognitive exercises simultaneously which may improve cognition of people with PD.

Another major focus in rehabilitation care is the advancement of using objective measures to quantify movement. Our group [33] and others [34, 35] have demonstrated that objective measures of balance and mobility are more sensitive to change compared to standard clinical measures after rehabilitation. Also, we know that the assessment of mobility in the clinic may not adequately reflect mobility during daily life, accurate measurements of walking and turning in daily life represent a technical challenge from both an algorithm standpoint and the storage of the large amount of data generated [36]. We recently demonstrated that quality, but not quantity, particularly turning and foot-strike angle, in daily life, is impaired in people with PD compared to similar age control participants [37, 38]. Here we will use wearable sensors worn during daily life to measure if Tele-ABC improves mobility in every day life. We hypothesize that gait quality measured during daily life will improve after balance telerehabilitation.

The present trial has three aims: I) *virtual assessment*: to determine if a virtual balance assessment (instrumented L-shape mobility test) with wearable sensors can predict a gold-standard clinical in-person assessment of balance, the Mini-BESTest; II) *virtual rehabilitation at home*: to explore the effects of 12 sessions of Tele-ABC (experimental group) and unsupervised-ABC (control group) on balance, gait, instrumented executive function tests, and clinical scales; and III) *home monitoring after telerehabiliation*: to explore if improvements after balance telerehabilitation transfer to daily-life mobility, measured by wearing instrumented socks for 7 days.

## Methods

### Trial design

**Fig. 1:**
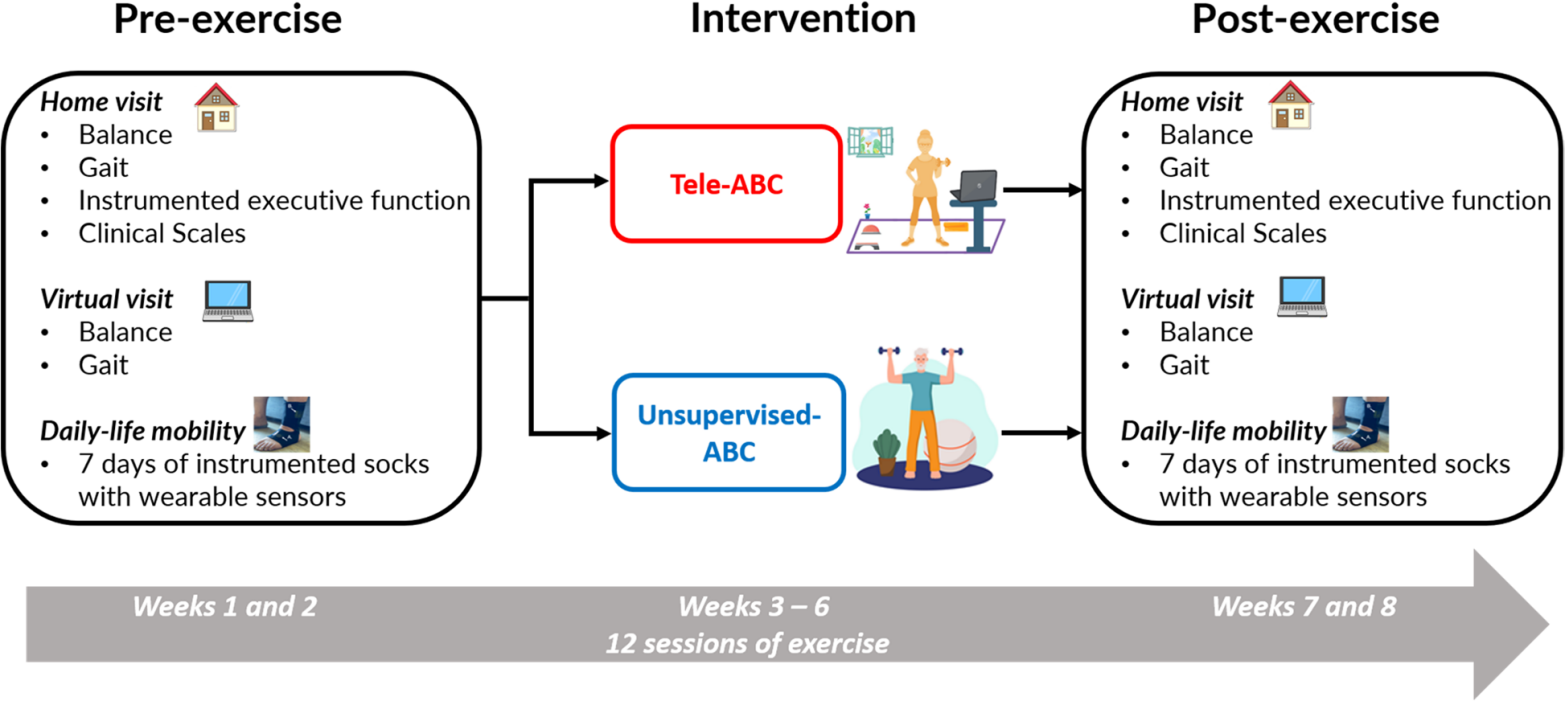
Study design. Abbreviation: ABC, Agility Boot Camp program

The Telerehabilitation Parkinson’s Disease (TelePD) Trial is a prospective, single-center, parallel-group, single-blind, controlled, randomized pilot trial. The study was approved by the Institutional Review Board at Oregon Health & Science University (OHSU) (eIRB #24,453) and will be conducted in accordance with the Declaration of Helsinki. This trial was prospectively registered on ClinicalTrials.gov (NCT05680597). This protocol paper follows the SPIRIT (Standard Protocol Items: Recommendations for Interventional Trials) 2013 statement and guidelines. The study design is depicted in Fig. 1.

### Participants and eligibility criteria

The target population for this study is people with PD who meet the following inclusion criteria for all aims of this trial: 1) diagnosis of idiopathic PD from a movement disorders neurologist with the United Kingdom Brain Bank criteria of bradykinesia and one or more of the following—resting tremor, rigidity, and balance problems not from visual, vestibular, cerebellar or proprioceptive conditions; 2) responsive to levodopa (self-reported); 3) Hoehn & Yahr stages I-III; 4) ages 55–85 years old; 5) ability to follow directions in order to participate in testing procedures and exercise sessions, 6) free of any medical conditions or medication that contraindicates participation in an exercise program and 7) willing and able to participate in 12 sessions of rehabilitation intervention while also refraining from changes to other exercise programs and medications during the study period, within reason.

Exclusion criteria are: 1) major musculoskeletal or neurological disorders, structural brain disease, epilepsy, acute illness or health history, other than PD, significantly affecting gait and balance (i.e., musculoskeletal disorder, vestibular problem, head injury, stroke, cardiac disease, etc.); 2) a medical condition that precludes exercise; 3) cognitive inability to participate in an exercise program, such as Montreal Cognitive Assessment score less than or equal to 19 [39], prior diagnosis of dementia or inability to follow directions, 4) recurrent fallers, defined as more than 3 falls per week (from the patient and caregiver recollection), 5) excessive use of alcohol or recreational drugs; 6) recent change in medication; 7) inability to stand and walk for instrumented L-shape mobility test.

### Recruitment, randomization, and allocation

Subjects will be recruited from the OHSU Movement Disorders Clinic, the OHSU Parkinson Center of Oregon and Movement Disorders clinical program, the Balance Disorders Laboratory, the Oregon Clinical & Translational Research Institute, local neurologists (at Portland Metro area hospitals and clinics), community recruitment (e.g., public talks and distribute study flyers at various community outreach events and locations, such as the Senior Living Centers, Athletic Clubs, Rotary Clubs, Parkinson Support groups).

This trial will enroll 80 eligible people with PD who will be randomized at a 1:1 ratio into the experimental group (*n* = 40) or control group (*n* = 40). Both groups will perform 12 sessions of exercise at home, with each session lasting 60 min, over approximately 4 weeks. Within 24 h after baseline testing the participant will be randomized using an online module in the Research Electronic Data Capture system (REDCap). The participant will learn their random group assignment after both enrollment and the baseline testing. The randomization module in REDCap allows the randomization of participants based on the Hoehn & Yahr stage. The module also monitors the overall allocation progress and assignment of randomized subjects. We used a stratified randomized block design to allocate participants to the either experimental or control group. Randomization to arms is balanced 1:1 for each stratum, Hoehn & Yahr I and II, and Hoehn & Yahr III, respectively. We additionally used randomly permutated blocks of 2, 4, and 6 in order to keep the randomization scheme blinded to the blinded research team members to help avoid selection bias. All subjects will agree not to change their exercise activities or antiparkinsonian medication during the duration of the study, unless necessary.

### Blinding

Due to the nature of the intervention, participants and physical therapists cannot be blinded to group allocation. However, participants will be blinded to the expected outcomes, unaware of the study hypotheses, and will be explicitly instructed not to discuss their training program with the assessors to maintain assessor blinding. A trained assessor who is blinded to group allocation will be responsible for all outcome assessments. Data analysts and statisticians will also be blinded to group allocation.

### Intervention

**Fig. 2:**
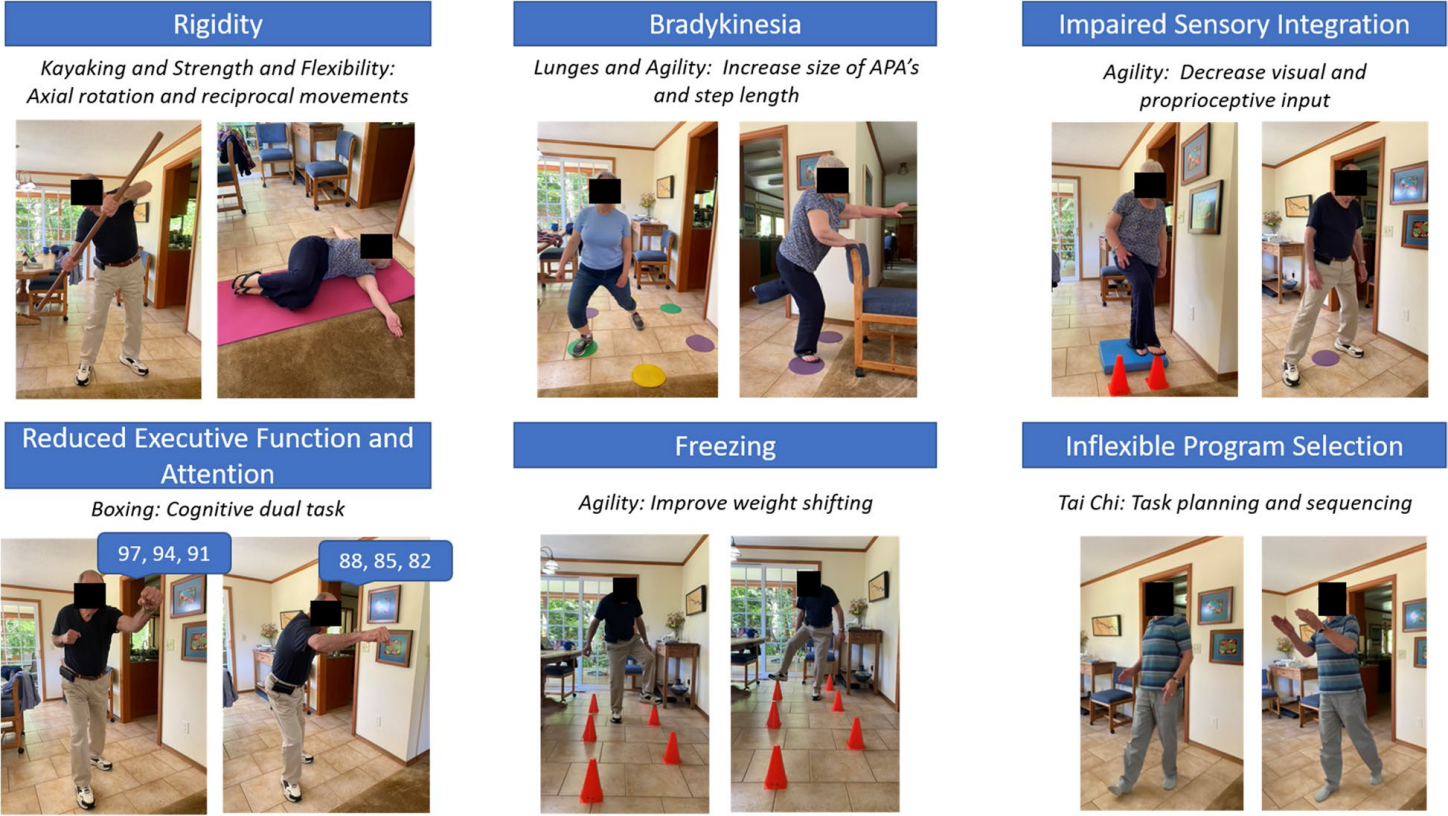
Exercise stations used in the exercise programs

The balance telerehabilitation intervention is based on the theoretical framework described in the ABC program for PD that we have previously published [28, 33, 40, 41]. Participants will be randomized at a 1:1 ratio into receiving home-based balance exercises in either balance telerehabilitation, called Tele-ABC (experimental group), or unsupervised exercises, called unsupervised-ABC (control group). Both groups (Tele-ABC and unsupervised-ABC) will receive identical exercises (Table 1) with 3 levels of progressions (Table 2) which the physical therapist will tailor to each person. Each participant will be instructed to exercise three times per week for 60 min for approximately 4 weeks. The exercise program consists of 6 exercise ‘stations’ (Fig. 2) with 3 levels of difficulty per task. Each exercise will be challenged with speed/amplitude increases, sensory challenges (eyes closed or soft surface), and/or cognitive (dual-task training) additions. For the unsupervised-ABC group, the first session will be conducted in person with the physcial therapist, which is common in clinical care – an in-person session to instruct in exercise followed by the person doing the exercises unsupervised at home. This is particularly true when people live in rural locations without easy access to physical therapy. Level of perceived exertion (0–10 scale) will be recorded for each station weekly by the physical therapist (experimental group—Tele-ABC) and in a paper journal (control group – unsupervised-ABC) [42]. The level of self-reported exertion will be recorded to determine the level of challenge of the program.

**Table 1:** Physiological constraints in Parkinson’s disease that affect balance and exercise

Constraint	Impact on balance and mobility	Exercise principles	Exercise examples
*Rigidity*	Flexed posture ↓Trunk rotation ↓Range of motion	↑Axial rotation ↑Reciprocal movements ↑Upright posture ↑Limits of stability ↑Big steps	Kayaking Shoulder roll
*Bradykinesia*	Small and slow movements ↓Base of support	↑Size of APA ↑Base of support	Clock lunges Speed skater
*Impaired Sensory Integration*	↓Kinesthetic awareness Overutilization of vision Instability on unstable surfaces	↓Use of visual and proprioceptive input ↑Kinesthetic awareness	Cone tap standing on foam and wearing sunglasses
*Reduced Executive Function and Attention*	↓ Performance with dual tasks ↓Ability for complex sequencing of actions	↑Performance with dual task (cognitive or motor)	Boxing with cognitive dual task Tai Chi
*Freezing*	↓APA ↓Visuospatial skills ↓Mobility with divided attention	↑Weight shifting ↓Use of visual and auditory cues Layer exercises with dual task	Footwork drills: progressing to wearing sunglasses and adding dual task
*Inflexible Program Selection: task planning and sequencing*	↓Performance on functional transfers: sit-to-stand, rolling, floor transfers ↓Ability to quickly change strategy	↑Performance of sequencing components of transfers ↑Ability to quickly change strategies	Floor exercises including rolling and getting on/off the floor

*Abbreviations*: *APA* Anticipatory postural adjustment

**Table 2:** Levels and progression of each exercise station

Exercise station	Level	Safety modifications	Sensory level	Speed level (metronome)	Dual-task level
**Tai Chi (10 min)**: prayer wheel, cat walk, cloud hands	1	UEs on counter/chair	None	None	None
	2	Hands hovering or near the wall	None	None	None
	3	No UE support	None	None	None
**Kayaking (5 min)**: basic stroke	1	Seated	Eyes open	Slow: 25 bpm	None
	2	Standing leaning against the counter	Eyes closed	Medium: 35 bpm	Easy level (cognitive^a^, motor^c^)
	3	Standing without support	Foam pad	Fast: 45 bpm	Hard level (cognitive^b^, motor^d^)
**Agility (15 min):** footwork drill, speed skater, toe taps	1	UE support on chair or counter	None	None	None
	2	No UE support	Sunglasses	None	Easy level (cognitive^a^, motor^e^)
	3	No UE support + movement	Foam pad	None	Hard level (cognitive^b^, motor^f^)
**Boxing (5 min)**: jab/cross/upper cut combo	1	Standing leaning against counter	Eyes open	metronome can be used	None
	2	Standing without support	Eyes closed	metronome can be used	Easy level (cognitive^a^)
	3	Standing without support + stepping	Foam pad	metronome can be used	Hard level (cognitive^b^)
**Lunges (5 min)**: clock stepping	1	UE support on chair or counter	Eyes open	metronome can be used	None
	2	No UE support	Sunglasses	metronome can be used	Easy level (cognitive^a^, motor^g^)
	3	No UE support + head rotation R/L with each step	Foam pad	metronome can be used	Hard level (cognitive^b^, motor^h^)
**Strength and Flexibility (15 min)**: sit to stand, bridge, shoulder release, cobra, trunk extension, cat camel	1	Bed	None	None	None
	2	Floor transfer with UE	None	None	None
	3	Floor transfer without UE support	None	None	None

*Abbreviations*: *UE* Upper extremities, *R* Right, *L* Left

^a^cognitive task: verbal fluency (e.g., naming animals, countries, fruits, girl/boy name) and serial subtraction (3 or 7 starting at 100)

^b^cognitive task: serial subtraction (3 or 7 starting at 5000), counting backwards, spelling backwards, saying alphabet backward, naming a girl/boy name for every letter of the alphabet, repeating back each name each time (e.g., A Annabel, A Annabel B Becky, A Annabel B Becky C Catthy, etc.)

^c^motor task: march in place

^d^motor task: march in place with knee kick

^e^motor task: ball toss/catch

^f^motor task: balance ball on the cone with movement

^g^motor task: reach in the direction of the step

^h^motor task: ball catch

### Safety issues

All adverse events, either during physical therapy or during data collection in the home and/or virtually will be recorded and reported. Falls and musculoskeletal strains are the greatest risks for exercise in patients with PD who have balance and gait impairments. An initial home visit by the physical therapist and customized levels of exercise progression and modification will help ensure safety in the home during exercise sessions.

### Assessment procedures

The pre-screening for inclusion/exclusion criteria will be done over the phone. All assessments will take place at the participant’s home and/or virtually. Immediately after the home and virtual assessments at baseline, participants will complete 7 days of daily-life mobility monitoring and then start the 12 intervention sessions (experimental or control). Following the 12 sessions, participants will complete post-testing repeating the home and virtual assessments and daily-life monitoring for 7 days. An overview of the study assessments is detailed in Table 3. All the participants will be assessed at the same time of day and in the clinically defined ‘‘on’’ state (fully medicated).

**Table 3:** Overview of the study assessments and intervention period

Outcome Assessments	Pre-exercise: Home Visit	Pre-exercise: Virtual Visit	Pre-exercise: 7 days of Sensors During Daily Life	12 sessions of exercise intervention	Post-exercise: Home Visit	Post-exercise: Virtual Visit	Post-exercise: 7 days of Sensors During Daily Life
Mini-BESTest *(primary outcome)*	X				X		
Instrumented L-shape mobility test with wearable sensors		X				X	
ISAW with wearable sensors	X				X		
Daily-life mobility with instrumented socks and wearable sensor			X				X
MDS-UPDRS	X				X		
ABC	X				X		
PDQ-39	X				X		
NFoGQ	X				X		
FES-I	X				X		
IPAQ	X				X		
5 times sit-to-stand task	X	X			X	X	
360-degree Turn Test (right and left)	X	X			X	X	
Floor Transfer Test	X				X		
Self-Efficacy for Exercise	X				X		
Life-Space Questionnaire	X				X		
TSS					X		
HEP					X		
TabCAT	X				X		
Timed-up-and-go test		X				X	
Self-perceived Mini-BESTest		X					
PGIC					X		

*Abbreviations*: *Mini-BESTest* Mini Balance Evaluation Systems Test, *ISAW* Instrumented Stand and Walk Test, *MDS*-*UPDRS* Movement Disorder Society-Sponsored Revision of the Unified Parkinson’s Disease Rating Scale, *ABC* Activities-Specific Balance Confidence, *PDQ-39* Parkinson’s Disease Questionaire-39, *NfoGQ* New Freezing of Gait Questionnaire, *FES-I* Falls Efficacy Scale-International, *IPAQ* International Physical Activity Questionnaire- Short Form, *TSS* Telerehabilitation Satisfaction Scale, *HEP* Home Exercise Program, *PGIC* Patient Global Impression of Change, *TabCAT* Tablet-based Cognitive Assessment Tool

### Home-based, in-person pre- and post-exercise

In the home, the following assessments will be performed: i) the Mini-BESTest, a 14-item test of balance shown to be related to fall risk in people with PD [43]; ii) ISAW with and without a cognitive task (serial subtraction by threes from a three-digit number) where participants will wear 6 Opal sensors (APDM Wearable Technologies, a Clario company) placed over feet, lumbar area, wrists, and sternum. Participants will be instructed to look straight ahead for 30 s, walk straight ahead for 7 m, turn 180 degrees, and return to the initial starting location; iii) The MDS-UPDRS I-IV to assess PD-related non-motor experiences of living, motor experiences of daily living, motor severity, and motor complications [44]; iv) the Activities-Specific Balance Confidence (ABC) scale to assess the participant’s perception of balance confidence [45]; v) the Parkinson's Disease Questionaire-39 (PDQ-39) to assess how PD affects the quality of life over the last month [46]; vi) the New Freezing of Gait Questionnaire (NFoGQ) to assess the severity of FOG [47]; vii) the Falls Efficacy Scale-International scale to assess fear of falling [48]; viii) the International Physical Activity Questionnaire-Short Form (IPAQ) to assess the intensity and type of physical activity performed in the last 7 days [49]; ix) the five times sit-to-stand task [50]; x) the 360 degrees Turn test [51]; xi) the floor transfer test [52]; xii) the Self-efficacy for exercise scale to assess the participation in exercise [53]; xiii) the Life-Space Questionnaire to assess mobility based on how far and how often a person travels inside and outside home space [54]; xiv) cognitive function assessment using the Tablet-based Cognitive Assessment Tool (TabCAT) tests on an iPAD to assess visuospatial and executive function (Benton Judgement of Line Orientation, Flanker test, and Set Shifting test) [55–57]. At the post-test assessment, participants will complete all assessments performed at the pre-test and also the Telerehabilitation Satisfaction Scale (TSS) to assess the overall satisfaction with the telerehabilitation program [15] and the Patient Global Impression of Change (PGIC) to assess the impression of change after intervention [58].

### Home-based virtual pre- and post-exercise

Virtually (using home computer, iPAD, or study computer) we will administer: i) an instrumented L-shape mobility test where participants will wear 3 sensors (one Opal sensor on a belt with the sensor placed over the lumbar area and two Opal instrumented socks by APDM Wearable Technologies, a Clario company, placed on both feet) and will be instructed to sit for 30 s, then stand with feet together and look straight ahead for 60 s, walk at least 10 steps before turning 90 degrees, and take at least 5 steps to complete an L-shaped path, turn 180 degrees, walk back and finish an L-shaped path. The participants will perform 5 repetitions of an L-shape path continuously, and end with 30 s of quiet sitting. This instrumented L-shape mobility test can characterize multiple domains of balance (*see details of balance outcome in *Table 4), similar to the Mini-BESTest, including postural sway in standing with feet together with eyes open for 30 s before step initiation and gait with a 180-degree turn [11]; ii) the five times sit-to-stand task [50]; iii) the 360 degrees Turn test [51]; and iv) the 3 m timed-up-and-go test [59]. Self-perceived Mini-BESTest, where the participant is asked to image how well they would perform on the balance tasks of the Mini-BESTest will be assessed only in the pre-exercise assessment.

**Table 4:** Objective balance and gait outcome measures collected at home with both virtual instrumented L-shape mobility test and daily-life mobility monitoring (7 days), divided by balance domains

*Balance and Gait Domains/Measures*	*Virtual instrumented L-shape mobility test, 3 wearable sensors*	*Daily-life mobility with passive monitoring, 3 wearable sensors*
Postural Sway		
Root Mean Square, Mediolateral	X	No
Root Mean Square, AnteroPosterior	X	No
Jerk, Mediolateral	X	No
Anticipatory Postural Adjustment (APAs)		
Peak Mediolateral	X	No
First Step Range of Motion	X	No
Gait: average		
Gait speed	X	X
Angle at Heel Strike	X	X
Stride length	X	X
Turning: Average		
Duration	X	X
Velocity	X	X
Angle	X	X
# Steps	X	X
Gait Variability		
Gait Speed	No	X
Angle at Heel Strike	No	X
Stride length	No	X
Swing Time	No	X
Duration	No	X
Velocity	No	X
Angle	No	X
# Steps	No	X

### Daily-life monitoring pre- and post-exercise

Participants will wear Opal Instrumented Socks that are made of a thin elastic cloth and have inertial sensors embedded for the measurement of mobility in daily life. When worn, the sensors are located on the dorsum of the foot with the battery above the lateral malleolus, as previously published [38]. They will also wear an Opal sensor on a belt with the sensor placed over the lumbar area (Opal and Opal instrumented socks by APDM Wearable Technologies, a Clario company). Participants will don the sensors in the morning and wear them for at least 8–10 h per day for 7 days. The sensors will be taken off and placed in a charger each night. At the end of the 7 days, the sensors will be returned by mail or picked up from their home by a study team member. See details of balance and gait outcomes in Table 4.

### Primary and secondary outcome measures

The Mini-BESTest will be the primary outcome, while objective balance and gait measures, daily-life mobility, instrumented executive function tests, and clinical scales described in Tables 4 and 5 will be secondary outcomes.

**Table 5:** Clinical and functional outcomes measures

*Name*	*Description*
Mini-BESTest	14-item assessment of 4 balance domains: anticipatory, reactive, sensory, and dynamic gait
ISAW with wearable sensors	Quiet stance for 30 s, walk 7 m, turn 180 degrees, and return to the initial location in single and dual tasks (normal speed)
MDS-UPDRS	Clinical scale of severity of non-motor experiences of daily living, motor experiences of daily living, motor examination, and motor complications
ABC	16-item self-report scale of confidence in performing daily activities
PDQ-39	39-item self-report questionnaire to assess Parkinson’s disease-specific health-related quality in eight domains of quality of life
NFoGQ	9-item self-report scale of freezing of gait severity
FES-I	16-item questionnaire to assess fear of falling
IPAQ	Short Form to assess the intensity and type of physical activity performed in the last 7 days
5 times sit-to-stand task	Time to complete 5 repetitions sit-to-stand as quickly as possible
360 Degree Turn Test (right and left)	Time to complete fast 360 degrees to turn left and right as quickly as possible
Floor Transfer Test	Time to transfer from standing to laying down on back on the floor as quickly as possible
Self-Efficacy for Exercise	9-item self-report scale of efficacy for exercise
Life-Space Questionnaire	5-item self-report scale of life-space level
TSS	15-item self-report scale of satisfaction with the telerehabilitation program
HEP	15-item self-report scale of satisfaction with the home-based exercise program
TabCAT	Instrumented executive function using an IPAD
Timed-up-and-go test	Rise from a chair, walk 3 m, turn 180 degrees, return to the chair, and sit down (normal speed)
Self-perceived Mini-BESTest	14-item self-perceived assessment of 4 balance domains: anticipatory, reactive, sensory, and dynamic gait
PGIC	Patient rating the impression of change after intervention

*Abbreviations*: *Mini-BESTest* Mini Balance Evaluation Systems Test, *ISAW* Instrumented Stand and Walk Test, *MDS*-*UPDRS* Movement Disorder Society-Sponsored Revision of the Unified Parkinson’s Disease Rating Scale, *ABC* Activities-Specific Balance Confidence, *PDQ-39* Parkinson’s Disease Questionaire-39, *NfoGQ* New Freezing of Gait Questionnaire, *FES-I* Falls Efficacy Scale-International, *IPAQ* International Physical Activity Questionnaire- Short Form, *TSS* Telerehabilitation Satisfaction Scale, *HEP* Home Exercise Program, *PGIC* Patient Global Impression of Change, *TabCAT* Tablet-based Cognitive Assessment Tool

### Data management and monitoring

Data entry will be handled by the team members. Data will be entered into REDCap and the OHSU Balance Disorders database. Hard copies of these records will be stored inside a locked office in a locked cabinet at OHSU. The de-identified Mobility Lab data will be collected on a password-protected and data-encrypted laptop computer and uploaded after each test session to an OHSU secure server, where the Balance Disorders database is located. The computer that will store the project database will be protected by current network security behind the OHSU firewall. All data will be double-checked for accuracy by a person who is not involved in the day-to-day life of the study.

### Sample size calculation

#### Aim 1

To estimate power and sample size for relating the instrumented L-shape mobility test collected virtually in the home to the Mini-BESTest in-person, we used the pilot data from 114 people with PD who had measures on the Mini-BESTest and the ISAW – both performed in-person in the Balance Disorders Laboratory. Using multiple linear regression, we used a subset of objective measures to predict the Mini-BESTest total score as well as the four Mini-BESTest domain sub-scores. The subset of objective measures was selected based on bivariate correlations between the objective measures and the Mini-BESTest total score. A correlation at or above 0.4 was retained and included: 1) gait speed, 2) angle of the foot at heel strike, 3) stride length, 4) turning duration, 5) turning velocity, 6) steps during turning, and 7) range of motion of the 1^st^ step. These objective measures accounted for 48% of the explained variance in the Mini-BESTest total score and between 26–40% in the four Mini-BESTest subscores. We used this range of R^2^ as the parameter in our power calculations. Power was estimated at alpha equal to 0.05 and a sample size of 80. We will have at least 97% power to detect R^2^ of 0.26 or greater.

#### Aim II

One of the goals of Aim II is to generate preliminary estimates of the sensitivity of balance measures (Mini-BESTest and ISAW) due to the Tele-ABC program. These estimates ultimately will be used to inform power and sample size computations for a larger clinical trial. We did examine effect sizes detectable by intervention at the planned sample size and related these to effect sizes observed in other similar interventions. In our pilot study, we observed improved dual-task gait speed using the same, wearable mobility sensors after an in-person balance exercise intervention [41]. We observed an effect size of 0.94 (95%CI 0.67, 1.21). Using the means and standard deviations from these data (0.79 m/s and 0.92 m/s (pre and post means); 0.20 (sd)), we estimated the power to detect a change in dual-task gait speed with our proposed sample size of 40 participants (experimental group) using a paired sample t-test. With alpha set to 0.05, we will have approximately 96% power to detect changes in gait speed in the experimental group. Although the intervention proposed here is relatively small and not designed to be a highly powered trial itself, we will make use of a randomized, two-group intervention design.

#### Aim III

One of the goals of Aim III is to generate preliminary estimates of effect size for daily life metrics measured in the home after rehabilitation, for both the groups (experimental and control). These estimates ultimately will be used to inform power and sample size computations for a larger clinical trial. Specifically, we will calculate effect size estimates from 5 main home metrics that are predicted to be the most likely to show improvement after rehabilitation (turn angle, swing variability [coefficient of variation], the angle at heel strike, the angle at heel strike variability [coefficient of variation], and stride length variability [coefficient of variation]). Using paired sample t-tests, we estimate that we will have at least 80% power to detect pre/post differences with moderate effect sizes (Cohen’s d = 0.45) or greater with 40 participants at an alpha level of 0.05.

### Statistical analysis

#### Aim I

To determine if a virtual, balance assessment (instrumented L-shape mobility test) using wearable sensors can predict a gold-standard, clinical in-person assessment of balance (Mini-BESTest), we will use multiple linear regression. We will fit linear regression models with the total Mini-BESTest score, in addition to the four Mini-BESTest domain sub-scores. To determine the optimal set of predictors, we will employ three separate analytic approached to select predictors: 1) assess predictors based on their individual contribution to total R^2^ while considering their statistical significance; 2) stepwise selection method in linear regression models; and 3) advanced statistical learning approaches such as best subset regression analysis, LASSO regression, and random forest classifiers. Subsequent models will be adjusted for relevant covariates. Adjustments will be performed for multicomparison purposes.

#### Aim II

The primary outcome, Mini-BESTest score, and its subscores, pre and post-exercise will be compared between the two groups. Secondary outcomes described in Tables 4 and 5 will also be examined. We will use three statistical procedures: 1) linear mixed models will be used having a group (experimental and control) and time (pre and post-exercise) as fixed factors with included interaction (group x time) to indicate a difference of changes in outcomes between the groups. Individual random effects will also be included. Adjustments will be used for multicomparison purposes. Analyses will first be conducted without including other covariates, but followed by models with covariates (e.g., age, gender, body mass index, disease severity, freezing of gait, and cognition) to account for potential confounding effects; 2) analysis of covariance on delta change controlling for baseline measures. Covariates will be included in the subsequent analysis; and 3) the pre-post change in outcomes after each intervention will be compared between groups via independent t-tests (no covariates) and effect size with confidence interval. The estimates from this pilot study will be used to inform the sample size for a future clinical trial.

#### Aim III

To determine if daily-life mobility changes after interventions, our main analysis will be the paired t-tests to compare the values in each metric described in Table 4 in the pre and post-exercises, separately. We will use the same statistical procedures as described in Aim II to explore improvement (estimated) between groups since no study has investigated the effects of any intervention on daily-life mobility using instrumented socks with wearable sensors.

Missing data: We will first assess the missing data frequency and patterns for both primary outcomes. If ≤ 5% of observations are missing the primary outcome, then multiple imputation methods will not be of benefit and we will perform analysis without imputation. If > 5% of observations are missing outcomes, we will perform multiple imputations as necessary to address the missing-ness. For all analyses, level of significance will be set at *p* ≤ 0.05.

## Discussion

Telerehabilitation is now commonly used for patients with PD, but there are several unknown aspects of its efficacy, both for the assessment and treatment of balance deficits in people with PD. In addition, studies on balance telerehabilitation (separate from virtual reality) for people with PD are scarce. For telerehabilitation to be effective, both assessment and treatment should be able to be completed virtually. The TelePD Trial targets both virtual assessment and treatment for balance problems in people with PD.

Regarding assessment, no virtual comprehensive balance assessment has been conducted pre and post-telerehabilitation in people with PD [18]. A gold standard clinical test is the BESTest [60], which evaluates balance across domains. The Mini-BESTest, a shorter version, is the most widely used balance assessment tool in physical therapy clinics and research protocols [43, 61]. While the Mini-BESTest, a clinical measure, has been studied extensively and for PD has been shown to be reliable and valid both for measuring multiple domains of balance [61], measuring balance domains in a virtual setting is unexplored. We recently published data showing that instrumented balance and gait tests using wearable sensor technology can successfully quantify different domains of balance and gait in older adults [11]. Both clinical (Mini-BESTest) and ISAW measure independent domains of balance and gait (Table 4). Thus, we will use wearable sensors to determine if we can predict Mini-BESTest scores (as the clinical, in-person, gold standard) using the instrumented L-shape mobility test to objectively and remotely quantify balance across domains in people with PD. As balance is particularly challenging to assess virtually, clinical maneuvers or sophisticated equipment are not available in people’s homes, our study will help determine if a simple but objective balance and gait assessment can be applied virtually in future telerehabilitation trials or for a virtual balance evaluation. Furthermore, patients with cognitive impairment may be less responsive to a virtual approach- both assessment and/or intervention.

Regarding treatment, effective balance rehabilitation requires precise targeting of the impaired domain in order to ultimately treat the underlying deficit in balance dysfunction [43, 60, 62, 63]. Although we have previously demonstrated improvements after ABC in-person rehabilitation [33], the potential for administering this PD-specific, constraint-focused program virtually is unknown. Further, it is unknown if improvements after rehabilitation carry over to improvements in daily-life mobility. Passive monitoring of mobility during daily life can provide important measures of turning and variability that are difficult to detect in regular clinical scales [36–38] and may be sensitive to change after rehabilitation. An important aspect of continuous, passive monitoring, is the ability to measure variability in performance over time, which has previously been shown to be sensitive to early versus moderate PD [37, 64–66], fall frequency [67], and mild cognitive impairment in PD [68]. Specifically, we found that turning variability over the day and week was higher in people with PD compared to healthy controls, and variability measures increased with disease severity. Although studies have quantified gait and turning in daily life with wearable technology [64, 69, 70], including an association with falls and cognition, the effects of telerehabilitation on daily-life mobility are unknown.

The TelePD trial is designed to determine the usefulness of using wearable sensor-based measures of balance and gait remotely to assess balance, the feasibility and efficacy of balance telerehabilitation in people with PD, and the translation of balance improvements after telerehabilitation to daily-life mobility. The successful implementation of the TelePD Trial will shed further light on balance telerehabilitation interventions for people with PD. The long-term goal of this project is to develop an effective, virtual balance assessment and evidence-based treatment that can be used in any older adult with balance impairments.

### Supplementary Information

**Additional file 1: Supplementary Figure.  **Example template of recommended content for the schedule of enrolment, interventions, and assessments.

## Data Availability

Data sharing is not applicable to this article as no datasets were generated or analyzed during the current study. The final results of this study will be published in peer-reviewed journals.

## Abbreviations

PD: Parkinson’s disease
TelePD: Telerehabilitation Parkinson’s Disease Trial
OHSU: Oregon Health & Science University
SPIRIT: Standard Protocol Items: Recommendations for Interventional Trials
Mini-BESTest: Mini Balance Evaluation Systems Test
ISAW: Instrumented Stand and Walk Test
MDS*-*UPDRS: Movement Disorder Society-Sponsored Revision of the Unified Parkinson's Disease Rating Scale
ABC: Activities-Specific Balance Confidence
PDQ-39: Parkinson's Disease Questionaire-39
REDCap: Research Electronic Data Capture system
NfoGQ: New Freezing of Gait Questionnaire
FES-I: Falls Efficacy Scale-International
IPAQ: International Physical Activity Questionnaire- Short Form
TSS: Telerehabilitation Satisfaction Scale
HEP: Home Exercise Program
PGIC: Patient Global Impression of Change
TabCAT: Tablet-based Cognitive Assessment Tool
