# Supplementary material for: Balance telerehabilitation and wearable technology for people with Parkinson’s disease (TelePD trial)
Source: BMC Neurol. 2023 Oct 13;23:368. doi: 10.1186/s12883-023-03403-3 (PMC10571293; doi:10.1186/s12883-023-03403-3)
Supplement: Supplementary file 1 — Additional file 1: Supplementary Figure. Example template of recommended content for the schedule of enrolment, interventions, and assessments. [file 12883_2023_3403_MOESM1_ESM.docx]

Supplementary Figure. Example template of recommended content for the schedule of enrolment, interventions, and assessments.

|  | **STUDY PERIOD** | | | | | | | | | |
| --- | --- | --- | --- | --- | --- | --- | --- | --- | --- | --- |
|  | **Enrolment** | **Allocation** | **Post-allocation** | | | | | | | |
| **TIMEPOINT**** | ***entry*** | **0** | ***t_1_*** | ***t_2_*** | ***t_3_*** | ***t_4_*** | ***t_5_*** | ***t_6_*** | ***t_7_*** | ***t_8_*** |
| **ENROLMENT:** |  |  |  |  |  |  |  |  |  |  |
| **Pre-screening for inclusion and exclusion criteria by phone** | X |  |  |  |  |  |  |  |  |  |
| **Eligibility screen** | X |  |  |  |  |  |  |  |  |  |
| **Informed consent** | X |  |  |  |  |  |  |  |  |  |
| ***Assignment*** | X |  |  |  |  |  |  |  |  |  |
| **Allocation** |  | X |  |  |  |  |  |  |  |  |
| **INTERVENTIONS:** |  |  |  |  |  |  |  |  |  |  |
| ***Tele-Agility Boot Camp*** |  |  |  |  |  |  |  |  |  |  |
| ***Unsupervised-Agility Boot Camp*** |  |  |  |  |  |  |  |  |  |  |
| **ASSESSMENTS:** |  |  |  |  |  |  |  |  |  |  |
| *Mini-BESTest*  *(primary outcome)* |  |  | X |  |  |  |  |  | X |  |
| *Instrumented L-shape mobility test with wearable sensors* |  |  | X |  |  |  |  |  | X |  |
| *ISAW with wearable sensors* |  |  | X |  |  |  |  |  | X |  |
| *Daily-life mobility with instrumented socks and wearable sensor* |  |  |  | X |  |  |  |  |  | X |
| *MDS-UPDRS* |  |  | X |  |  |  |  |  | X |  |
| *ABC* |  |  | X |  |  |  |  |  | X |  |
| *PDQ-39* |  |  | X |  |  |  |  |  | X |  |
| *NFoGQ* |  |  | X |  |  |  |  |  | X |  |
| *FES-I* |  |  | X |  |  |  |  |  | X |  |
| *IPAQ* |  |  | X |  |  |  |  |  | X |  |
| *5 times sit-to-stand task* |  |  | X |  |  |  |  |  | X |  |
| *360 Degree Turn Test (right and left)* |  |  | X |  |  |  |  |  | X |  |
| *Floor Transfer Test* |  |  | X |  |  |  |  |  | X |  |
| *Self-Efficacy for Exercise* |  |  | X |  |  |  |  |  | X |  |
| *Life-Space Questionnaire* |  |  | X |  |  |  |  |  | X |  |
| *TSS* |  |  |  |  |  |  |  |  | X |  |
| *HEP* |  |  |  |  |  |  |  |  | X |  |
| *TabCAT* |  |  | X |  |  |  |  |  | X |  |
| *Timed-up-and-go test* |  |  | X |  |  |  |  |  | X |  |
| *Self-perceived Mini-BESTest* |  |  | X |  |  |  |  |  |  |  |
| *PGIC* |  |  |  |  |  |  |  |  | X |  |

**Abbreviations:** *t* = week; Mini-BESTest = Mini Balance Evaluation Systems Test; ISAW = Instrumented Stand and Walk Test; MDS-UPDRS = Movement Disorder Society-Sponsored Revision of the Unified Parkinson's Disease Rating Scale; ABC = Activities-Specific Balance Confidence; PDQ-39 = Parkinson's Disease Questionaire-39; NFoGQ = New Freezing of Gait Questionnaire; FES-I = Falls Efficacy Scale-International; IPAQ = International Physical Activity Questionnaire- Short Form; TSS = Telerehabilitation Satisfaction Scale; HEP = Home Exercise Program; PGIC = Patient Global Impression of Change; TabCAT = Tablet-based Cognitive Assessment Tool.
